# Carbonic Anhydrases III and IV Autoantibodies in Rheumatoid Arthritis, Systemic Lupus Erythematosus, Diabetes, Hypertensive Renal Disease, and Heart Failure

**DOI:** 10.1155/2012/354594

**Published:** 2012-09-16

**Authors:** Chengeng Liu, Yue Wei, Jianmin Wang, Langan Pi, Jianjun Huang, Peichang Wang

**Affiliations:** ^1^Department of Clinical Laboratory, Xuanwu Hospital, Capital Medical University, 45 Changchun Road, Beijing 100053, China; ^2^Department of Oncology, Dongfang Hospital, Beijing University of Chinese Medicine, Beijing 100078, China; ^3^Department of Clinical Immune, Xiangya School of Medicine, Central South University, Changsha 410078, Hunan, China

## Abstract

In the present study, the CA III and IV autoantibodies, CA activity, antioxidant enzymes and cytokines in rheumatoid arthritis (RA), systemic lupus erythematosus (SLE), diabetes, hypertensive renal disease, and heart failure were investigated. The anti-CA III antibody titers in patients with RA, SLE, and type 1 diabetes (T1D) were significantly higher than that in control groups (*P* < 0.05). The anti-CA IV antibody titers in patients with RA, SLE, type 1 diabetic nephropathy (T1DN), and heart failure were significantly higher than that in control groups (*P* < 0.05) while anti-CA IV antibody could suppress the total CA activity. The SOD and GPx levels in patients with RA, SLE, and T1DN were significantly lower than that in control groups (*P* < 0.05). IL-6, IL-17, IFN-**γ**, and TNF-**α** levels were significantly higher in SLE group compared with the control group (*P* < 0.05). Weak but significant correlations were found between anti-CA III antibodies and ESR in RA (*r* = 0.403, *P* = 0.013) and SLE patients (*r* = 0.397, *P* = 0.007). These results suggested that the generation of CA III and IV autoantibodies, antioxidant enzymes, and cytokines might influence each other and CA autoantibodies might affect the normal physiology function of CA.

## 1. Introduction

Autoimmune diseases arise from an inappropriate immune response of the body against substances and tissues normally present in the body. If the immune system mistakes some parts of the body or some proteins free in body fluid as deleterious substances, the immune system will produce specific antibodies to attacks it. This may be restricted to certain organs or involve a particular tissue in different places. The mechanism of autoantibodies formation which is the most critical part of autoimmune diseases is still not fully clear [1]. The autoantibodies are found in some disease which is not usually defined as autoimmune disease such as heart failure [2]. Lots of autoantibodies are detected in many kinds of diseases, but only dozens of them are used in the clinical diagnosis and/or therapy monitoring. The relationship between the new-found autoantibodies and other clinical indicators needed to be evaluated systemically.

Carbonic anhydrases (CA, EC 4.2.1.1) are zinc-containing enzymes, which play a critical role in maintaining the intercellular/extracellular pH of most mammalian cells by catalyzing the interconversion between carbon dioxide and bicarbonate. CA is a group of widespread metalloenzymes and there are at least 15 different isoforms present in mammalian cells [3]. Some of these isozymes are membrane-bound enzymes (CA IV, CA IX, and CA XII CA XIV, etc.), whereas others are located in the cytosol (CA I, CA II, CA III, CA VII, CA XIII), CA V is mitochondrial and CA VI is secreted in the saliva and milk. Autoantibodies response to CA I, CA II, and CA IV have been found in the patients with rheumatoid arthritis (RA) and autoimmune pancreatitis (AIP) which are autoimmune diseases in the conventional sense [4–6].

CA III is a cytoplasmic enzyme that exhibits a relatively low carbon dioxide hydratase activity. It is expressed at a very high level in skeletal muscle, where physical exercise has been shown to increase free radical production. CA III may play a role in scavenging oxygen radicals and thereby protecting cells from oxidative damage [7, 8]. In addition, CA III has been demonstrated to have a carboxyl esterase activity and phosphatase activity, which suggests that it is a tyrosine phosphatase [7]. In kidney, CA IV is present on the apical brush-border membrane and on the basolateral membrane of proximal tubule cells, which contributes to net transepithelial bicarbonate transport [9, 10]. Most cardiac CA appears to be bound to SR and sarcolemmal membranes while carbonic anhydrase IV is the predominant isozyme in the heart [11].

Circulating auto-antibodies have been critically linked to several kinds of diseases. Their prevalence, mode of action, and potential therapeutic modulation are intensively investigated. In the present study, we determined the antibodies response to the CA III and CA IV in the serum of Chinese patients with RA, systemic lupus erythematosus (SLE), type 1 diabetes (T1D), type 1 diabetic nephropathy (T1DN), type 2 diabetes (T2D), type 2 diabetic nephropathy (T2DN), hypertensive nephropathy, and heart failure using indirect enzyme-linked immunosorbent assay (ELISA) and investigated the possible associations between these antibodies and other indicators of these diseases.

## 2. Materials and Methods

### 2.1. Study Population

The design of this study was approved by the ethics committee of Xuanwu hospital of Capital Medical University, informed consent was obtained from all participants. 91 RA patients which did not receive immunosuppressive treatment for at least 1 year (57 females, 34 males, mean age 52.5 ± 11.8), 79 SLE patients which did not receive immunosuppressive treatment for at least 1 year (56 females, 23 males, mean age 32.5 ± 9.8), 157 T1D patients (90 females, 67 males, mean age 25.5 ± 8.7), 56 T1DN patients (30 females, 26 males, mean age 32.5 ± 11.8), 188 T2D patients (107 females, 81 males, mean age 62.5 ± 13.7), 65 T2DN patients (32 females, 33 males, mean age 65.5 ± 8.6), 165 hypertensive nephropathy patients (84 females, 81 males, mean age 68.4 ± 9.5), and 73 heart failure patients (25 females, 48 males, mean age 70.6 ± 7.4) were selected for this study. Age- and gender-matched control subjects were also included. Serum samples were stored at −80°C until analysis.

The following data of the subjects were determined using automatic analyzer: rheumatoid factor (RF) and ASO are measured by Dade Behring BN II using immunonephelometric method; erythrocyte sedimentation rate (ESR) was measured using Vacuette ESR System; high sensitive C-reactive protein (hsCRP) and SOD activity were measured using Hitachi 7600 biochemical analyzer. The quality control products were tested with specimen.

The activity of catalase (CAT) and GPx and the level of total antioxidant eaPaeity (TAC), CPx, and MDA in serum were determined following the kit instructions (BioVision). Anticyclic citrullinated peptide antibodies (anti-CCP) were measured by ELISA kit from Euroimmun Medical Laboratory Diagnostics. IL-6, IL-10, IL-17, were measured by ELISA kit from R&D Corporation. TNF-*α* and IFN-*γ* were measured by ELISA kit from Invitrogen Corporation.

### 2.2. Detection of IgG Anti-CA III and Anti-CA IV by ELISA

Specific antibodies to CA III and CA IV (Sigma) were identified in serum by an indirect ELISA method. To conduct the assay, 100 *μ*L of the CA III (3.0 *μ*g/mL) and CA IV (2.5 *μ*g/mL) was incubated in an ELISA plate (Corning) at 4°C overnight. Microwells were then washed with phosphate-buffered saline (PBS: 0.01 M, pH 7.4) with 0.05% Tween-20 (PBST). Unbound sites were blocked by incubation with 200 *μ*L 20% newborn calf serum (NCS) in PBS at 37°C for 1.5 h. Sera were diluted 1 : 200 in blocking buffer and aliquots of 100 *μ*L were added to the wells. Wells coated with bovine serum albumin (BSA) were prepared for each sample, to assess nonspecific binding. After incubation at 37°C for 1 h, plates were washed 3 times with PBST. Subsequently, the captured antibodies were detected by a horseradish peroxidase- (HRP-) conjugated goat anti-human IgG (1 : 10000), Santa Cruz, which was diluted with 20% NCS in PBST (100 *μ*L/well). After incubation at 37°C for 30 min, wells were washed 5 times with PBST. Color was developed by application of 100 *μ*L of tetramethylbenzidine (Sigma) at 37°C for 20 min. The reaction was stopped by addition of 0.5 M sulfuric acid, and the optical density at 450 nm (OD_450_), with 620 nm as the correction wavelength, was obtained using an ELISA plate reader (Thermo MK3). Each sample was assayed in duplicate. A positive serum sample was included in each assay and used to correct for interassay variations. Results were expressed as arbitrary units (AU) calculated as ([OD_450_ of sample − OD_450_ of the nonspecific binding of the sample]/[OD_450_ of the positive control − OD_450_ of the nonspecific binding of the positive control]) × 100 [12, 13].

### 2.3. Determination of CA Activity

Erythrocyte CA hydratase activity was determined using potentiometric method [13, 14]. 100 *μ*L of serum was added to 2.4 mL of HEPES (pH = 8.80). The reaction was started by the addition of 2.5 mL ice-cold distilled water saturated with CO_2_. The reaction tubes were kept in ice bath. The rate of fall in pH from 8.2 to 7.0 was monitored continually with pH meter (Orion 420C-81). Rates of uncatalyzed CO_2_ hydration were subtracted from enzyme catalyzed ones. The enzyme unit (EU) of CO_2_ hydratase activity was calculated by using the equation EU = (*to*−*tc*)/*tc* where “*to*” and “*tc*” are the times for pH change of the nonenzymatic and enzymatic reactions, respectively. CA activity was expressed as EU/mL. For suppression experiment, the CA IV antibody (final concentration was 1 : 100) was added into the reaction system and then determined whether CA activity was suppressed (*n* = 5). Each sample was assayed in triplicate.

### 2.4. Statistical Analyses

Statistical analyses were performed using SPSS 13.0 for Windows. For normally distributed data, results are expressed as the mean and standard deviation (SD); differences between groups were assessed by *t*-tests. Differences between groups were analyzed using the Mann-Whitney *U-*test while correlations were determined by computing Spearman rank correlation coefficients. *P* values of less than 0.05 were considered as significantly different.

## 3. Results

The anti-CA III antibody titers in patients with RA, SLE, and T1DN were significantly higher than that in control group (*P* < 0.05). The anti-CA III antibody titer of hypertensive nephropathy patients group was a little higher (*P* > 0.05) compared with control group.

The anti-CA IV antibody titers in patients with RA, SLE, T1DN, and heart failure were significantly higher than that in control group (*P* < 0.05). The anti-CA IV antibody titer in patients with T2DN was a little higher (*P* > 0.05) compared with control groups.

**Figure 1:**
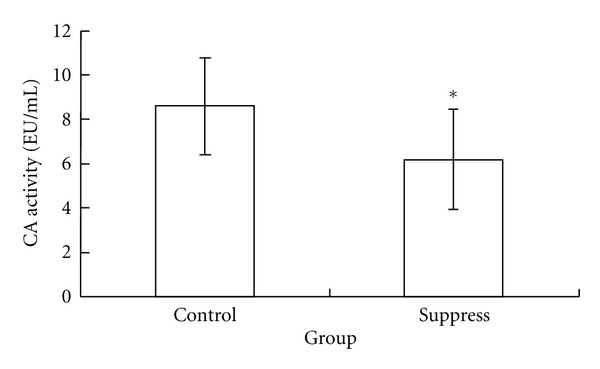
The total CA activity was suppressed by anti-CA IV antibody. *There was significant difference between suppress group and control group (*n* = 5) (*P* < 0.05).

The CA activity of RA, SLE, T1DN, T2DN, heart failure, and hypertensive nephropathy was significantly higher than that in the control groups, respectively (*P* < 0.05, Table 1). The CA activity could be suppressed by 1 : 100 anti-CA IV antibodies (*P* < 0.05, Figure 1).

Table 2 showed that the sera RF, ASO, and ESR in RA, SLE, and T1DN groups were significantly higher than that in control group, respectively (*P* < 0.05). The positive rates of ESR in RA and SLE were significantly higher than other patients groups (*P* < 0.05). A few of these indicators were higher in T1D patients and T2DN patients group compare to the control group (*P* < 0.05) but still in the in biological reference interval range.

The SOD and GPx levels in the serum of patients with RA, SLE, and T1DN were significantly lower than that in control groups, respectively (*P* < 0.05). There was at least one of these antioxidant enzymes significantly reduced in the T1D, T2DN, and hypertensive nephropathy compared with control group (*P* < 0.05). There was significant difference of MDA level between RA, SLE, T1DN, and heart failure groups compared with control groups, respectively (*P* < 0.05). The MDA level in the T1D, T2DN, and hypertensive nephropathy groups were little higher than control groups but there was no significant difference (*P* > 0.05, Table 3).

IL-6, IL-17, and IFN-*γ* levels were significantly higher in RA group compared with the control group (*P* < 0.05). IL-6, IL-17, IFN-*γ*, and TNF-*α* levels were significantly higher in SLE group compared with the control group (*P* < 0.05). IL-6 levels were significantly higher in T1DN, T2D, and T2DN groups compared with the control groups (*P* < 0.05). There was no significant difference of these cytokine between heart failure and hypertensive nephropathy patients groups and control groups, respectively (*P* > 0.05, Table 4).

Weak but significant correlations were found between anti-CA III antibodies and ESR in RA (*r* = 0.403, *P* = 0.013) and SLE patients (*r* = 0.397, *P* = 0.007).

## 4. Discussion

CA III is abundantly expressed in fat, liver, and slow-twitch skeletal muscle fibers [15]. The activity of CA III in hydrating carbon dioxide is lower than CA I and CA II, while it has two reactive sulfhydryl groups, which can reversibly conjugate to GSH through a disulfide bond [16, 17]. This s-glutathionylation reaction is believed to be an important component of cellular defense mechanisms that prevent the protein oxidation which is irreversible. In the present study, we found that the anti-CA III antibody titers in patients with RA, SLE, and T1DN were significantly higher than that in control group, respectively, while at least one of the antioxidant enzyme activity levels of MDA level in the serum of RA, SLE, T1D, T1DN, and T2DN patients was significantly lower than that in control group. The results were similar with the researches which take RA and diabetes patients as subjects [18, 19]. It has been proven that anti-CA II antibody was 25% higher in T1D patients than the controls [13]. In the present study, we found anti-CA III antibody and anti-CA IV antibody were positive in T1D and T2D patients.

Redox reactions are imperative to preserving cellular metabolism yet must be strictly regulated. Imbalances between reactive oxygen species (ROS) and antioxidants can initiate oxidative stress, which without proper resolve, can manifest into disease. Oxidative stress occurs when the generation of ROS overcomes the scavenging abilities of antioxidants. Such instances may be mediated by genetic lack of antioxidant enzymes as well as other triggers [20, 21]. T1D and T2D are frequently associated with increased oxidative stress [22]. The downregulation of antioxidant enzymes might be a trigger of autoimmune [13]. While the data from experiments using cell lines and animal models suggested that CA III might function to protect cells from oxidative damage [7, 22]. In this point of view, the anti-CA III antibody might be the trigger of the downregulation of antioxidant enzymes.

There are at least twenty autoantibodies associated with heart failure and cardiac dysfunction, including anti-Na pump, anti-SERCA, antilaminin and anti-hsp60 antibodies [2]. The injury to myocardium is believed to be a crucial trigger to the autoimmune of these two heart diseases. In this study, we found that the anti-CA IV antibody titer in the serum of heart failure patients was significantly higher than that of healthy control. The CA IV is the predominant CA isozyme in the heart, antigen exposure because of the long-time injury of heart cells might be the trigger of the generation of anti-CA IV antibody in heart failure patients [2, 11]. There was no significant change of the anti-CA IV antibody in the serum of patients with hypertensive nephropathy and T2DN, while total CA activity of these two diseases was higher than the control. It was suggested that the antigen exposure was not the trigger of the generation of anti-CA IV antibody in these diseases.

The cytokine including IL-6, IL-17, IFN-*γ*, and TNF-*α* were upregulated in these diseases. Overproduction of TNF-*α* supports and even amplifies the inflammatory process leading to insulin resistance [23]. TNF-*α* may activate both proapoptotic and antiapoptotic pathways. IL-17 plays an important role in the pathogenesis of several autoimmune diseases, and the importance of IL-17 has been demonstrated in various animal models [24, 25]. IL-6 has also been involved in metabolism, endocrine, and neoplastic disorders, and the endocrinopathy is also a motivator of autoimmune [2, 26].

Autoantibodies play important roles during physiological and pathological processes. Mouse models have shown that autoantibodies can activate the alternative pathway and induce in cell lysis and tissue damage [25]. In this study, we found that the anti-CA IV antibody could suppress the total CA activity. The mechanism of this phenomenon might be the active centre of CA IV was suppressed by the binding of CA IV and anti-CA IV antibody. This experiment was not done using anti-CA III antibody because the activity of CA III in hydrating carbon dioxide is very low (only about 2% of CA I and CA II [27]).

In conclusion, we found that there are unusually high titers of anti-CA III and/or anti-CA IV antibodies in the serum of RA, SLE, T1D, T1DN, T2D, T2DN, and heart failure patients with the abnormal level of cytokines and antioxidant enzymes. The generation of CA III and IV autoantibodies, the antioxidant enzymes, and cytokines might influence each other and the CA autoantibodies might affect the normal physiology function of CA.

## Figures and Tables

**Table 1:** The anti-CA III, IV titers, and the CA activity of subjects (mean (SD)).

		RA	SLE	T1D	T1DN	T2D	T2DN	HTN	HT
anti-CA III (abs)	control	0.058 (0.015)	0.056 (0.021)	0.064 (0.015)	0.041 (0.009)	0.053 (0.012)	0.064 (0.013)	0.049 (0.010)	0.061 (0.012)
	patients	0.195* (0.079)	0.327* (0.644)	0.073 (0.022)	0.204* (0.083)	0.065 (0.023)	0.056 (0.019)	0.096 (0.057)	0.065 (0.025)
	positive	11.2%	14.8%	0.0%	6.7%	0.0%	0.0%	0.0%	0.0%

anti-CA IV (abs)	control	0.021 (0.013)	0.032 (0.013)	0.027 (0.006)	0.019 (0.007)	0.026 (0.009)	0.027 (0.011)	0.035 (0.012)	0.026 (0.009)
	patients	0.183* (0.063)	0.205* (0.688)	0.033 (0.013)	0.022* (0.105)	0.022 (0.006)	0.049 (0.018)	0.028 (0.011)	0.131* (0.045)
	positive	9.8%	10.6%	0.0%	4.2%	0.0%	0.0%	0.0%	5.3%

CA activity (EU/mL)^†^	control	8.65 (2.86)	7.96 (3.12)	8.12 (2.57)	8.56 (2.13)	8.63 (2.64)	8.47 (2.32)	7.98 (3.65)	8.12 (2.43)
	patients	10.37* (4.36)	12.48* (5.68)	8.66 (4.36)	13.07* (5.58)	9.32 (3.97)	12.65* (4.56)	9.65* (4.51)	13.47* (5.08)

*There were significant differences between patients and control (*P* < 0.05).

^†^ *n* = 10 (5 males and 5 females).

HTN: hypertensive nephropathy; HT: heart failure.

**Table 2:** The disease indicators of subjects (mean (SD)).

		RA	SLE	T1D	T1DN	T2D	T2DN	HTN	HT
RF (U/mL)	control	7.6 (5.2)	5.8 (3.5)	6.9 (4.3)	7.2 (4.3)	7.5 (3.4)	8.1 (3.9)	7.7 (3.1)	6.7 (4.1)
	patients	108.4* (51.9)	63.8* (7.9)	11.4 (5.6)	12.3* (5.3)	9.1 (4.8)	10.5* (6.7)	8.2 (4.1)	7.5 (3.2)
	positive	83.2%	50.3%	3.9%	5.1%	6.4%	5.8%	2.8%	2.1%

ASO (IU/mL)	control	133.1 (23.2)	118.5 (31.4)	115.8 (21.7)	113.6 (35.7)	129.5 (41.5)	123.4 (43.7)	141.3 (32.4)	135.4 (39.8)
	patients	151.6* (45.5)	135.4* (42.3)	125.6 (33.9)	154.2* (36.8)	123.8 (41.6)	139.8 (37.9)	133.1 (45.8)	128.3 (21.5)
	positive	7.8%	6.5%	6.3%	7.2%	4.1%	5.9%	3.8%	2.2%

Anti-CCP (U/mL)	control	2.6 (0.7)	3.1 (0.8)	ND	ND	ND	ND	ND	ND
	patients	63.7 (33.5)	17.0 (5.9)	ND	ND	ND	ND	ND	ND
	positive	61.2%	11.2%	ND	ND	ND	ND	ND	ND

ESR, (mm/h)	control	ND	ND	ND	ND	ND	ND	ND	ND
	patients	41 (15)	37 (9)	18 (7)	17 (9)	15 (6)	19 (5)	11 (3)	11 (4)
	elevated	79.2% ^†^	65.1% ^†^	5.5%	3.5%	1.7%	2.4%	1.3%	1.9%

hsCRP (mg/L)	control	1.8 (0.7)	2.1 (0.9)	2.3 (0.7)	1.8 (0.5)	2.4 (0.5)	3.2 (1.2)	2.9 (1.3)	2.8 (2.1)
	patients	12.8* (5.7)	16.5* (8.2)	3.1 (1.2)	6.3* (0.8)	2.3 (0.8)	3.7 (1.5)	4.4 (1.5)	5.1 (1.9)
	elevated	35.8%	42.5%	3.9%	4.8%	5.1%	5.5%	7.8%	6.7%

*There were significant differences between patients and control (*P* < 0.05).

^†^There were significant differences between T1D, T1DN, T2D, T2DN, HTN, and HT groups.

HTN: hypertensive nephropathy; HT: heart failure; ND: not determined.

**Table 3:** The antioxidant enzyme activity and MDA of subjects (mean (SD)).

		RA	SLE	T1D	T1DN	T2D	T2DN	HTN	HT
SOD (U/mL)	control	134.57 (13.13)	151.55 (14.97)	142.83 (13.98)	145.66 (17.97)	139.63 (22.80)	141.10 (21.57)	149.00 (18.92)	142.13 (16.30)
	patients	92.85* (36.87)	105.60* (33.55)	131.19 (23.98)	133.68* (31.36)	131.69 (43.67)	115.37* (38.56)	131.69* (42.36)	101.93* (36.34)

GPx (U/mL)	control	158.18 (34.41)	171.20 (45.60)	162.68 (42.02)	159.67 (37.94)	164.36 (41.25)	155.39 (44.98)	157.65 (39.86)	159.36 (41.23)
	patients	118.29* (40.30)	135.85* (41.97)	146.58* (39.23)	127.96* (43.63)	159.67 (34.76)	161.33 (45.68)	149.35 (48.64)	135.36* (45.39)

CAT (U/mL)	control	1.42 (0.45)	1.56 (0.37)	1.54 (0.55)	1.39 (0.36)	1.74 (0.45)	1.61 (0.47)	1.52 (0.51)	1.73 (0.41)
	patients	1.33 (0.56)	1.37 (0.53)	1.39 (0.59)	1.11* (0.42)	1.25* (0.51)	1.33* (0.51)	1.47 (0.31)	1.39* (0.63)

TAC (U/mL)	control	14.31 (5.13)	15.12 (3.35)	15.65 (3.63)	14.96 (4.32)	15.69 (3.36)	16.01 (4.51)	14.63 (3.39)	14.78 (4.13)
	patients	11.25* (4.56)	13.59 (6.65)	14.32 (4.13)	12.15* (5.30)	14.34 (4.69)	14.96 (6.66)	13.97 (4.63)	12.01* (5.22)

MDA (nmol/mL)	control	3.57 (1.01)	3.31 (0.94)	3.14 (1.02)	3.46 (1.32)	3.35 (0.85)	3.61 (0.98)	3.44 (1.13)	3.57 (1.20)
	patients	4.52* (1.25)	4.29* (1.37)	3.08 (1.33)	4.05* (1.25)	3.45 (1.09)	3.96 (1.23)	3.55 (1.15)	4.11* (0.87)

*There were significant differences between patients and control (*P* < 0.05).

HTN: hypertensive nephropathy; HT: heart failure.

**Table 4:** The cytokine levels of subjects (mean (SD)).

		RA	SLE	T1D	T1DN	T2D	T2DN	HTN	HT
IL-6 (ng/L)	control	2.31 (0.63)	2.28 (0.71)	2.39 (0.69)	2.25 (0.67)	2.01 (0.58)	1.97 (0.64)	2.33 (0.59)	2.18 (0.61)
	patients	5.02* (1.41)	4.38* (1.64)	2.47 (0.96)	3.05* (1.53)	2.78* (0.88)	2.85* (0.93)	2.58 (1.22)	2.35 (0.97)

IL-17 (ng/L)	control	1.24 (0.21)	1.26 (0.29)	1.36 (0.31)	1.38 (0.27)	1.25 (0.33)	1.33 (0.26)	1.39 (0.40)	1.28 (0.35)
	patients	1.96* (0.90)	2.13* (0.75)	1.55 (0.76)	1.97* (0.65)	1.41 (0.57)	1.48 (0.73)	1.27 (0.70)	1.35 (0.55)

TNF-*α* (ng/L)	control	12.37 (2.45)	11.97 (3.69)	12.39 (3.21)	13.17 (3.39)	12.02 (4.13)	13.39 (3.64)	12.47 (3.68)	12.63 (4.36)
	patients	14.65 (6.34)	16.97* (6.93)	13.23 (5.61)	14.37 (8.35)	15.23* (4.32)	16.37* (6.55)	11.95 (4.37)	12.96 (3.37)

IFN-*γ* (ng/L)	control	11.01	12.25	11.69	12.64	11.64	10.89	11.39	12.43
	patients	16.38* (6.36)	14.99* (4.08)	12.06* (4.11)	14.15* (5.62)	12.03 (4.21)	11.59 (3.03)	12.78 (4.32)	13.36 (5.69)

*There were significant differences between patients and control (*P* < 0.05).

HTN: hypertensive nephropathy; HT: heart failure.
